# Infection with the SARS-CoV-2 B.1.351 variant is lethal in aged BALB/c mice

**DOI:** 10.1038/s41598-022-08104-4

**Published:** 2022-03-09

**Authors:** Fumihiko Yasui, Yusuke Matsumoto, Naoki Yamamoto, Takahiro Sanada, Tomoko Honda, Tsubasa Munakata, Yasushi Itoh, Michinori Kohara

**Affiliations:** 1 Department of Microbiology and Cell Biology, Tokyo Metropolitan Institute of Medical Science, 2-1-6, Kamikitazawa, Setagaya-ku, Tokyo, 156-8506 Japan; 2 Division of Pathogenesis and Disease Regulation, Department of Pathology, Shiga University of Medical Science, Otsu, Shiga 520-2192 Japan

**Keywords:** Immunology, Microbiology, Respiratory tract diseases, Pathogenesis, Infection, Inflammation

## Abstract

Models of animals that are susceptible to severe acute respiratory syndrome coronavirus 2 (SARS-CoV-2) infection can usefully evaluate the efficacy of vaccines and therapeutics. In this study, we demonstrate that infection with the SARS-CoV-2 B.1.351 variant (TY8-612 strain) induces bodyweight loss and inflammatory cytokine/chemokine production in wild-type laboratory mice (BALB/c and C57BL/6 J mice). Furthermore, compared to their counterparts, BALB/c mice had a higher viral load in their lungs and worse symptoms. Importantly, infecting aged BALB/c mice (older than 6 months) with the TY8-612 strain elicited a massive and sustained production of multiple pro-inflammatory cytokines/chemokines and led to universal mortality. These results indicated that the SARS-CoV-2 B.1.351 variant-infected mice exhibited symptoms ranging from mild to fatal depending on their strain and age. Our data provide insights into the pathogenesis of SARS-CoV-2 and may be useful in developing prophylactics and therapeutics.

## Introduction

Although more than a year and a half has passed since the outbreak of the novel coronavirus disease 2019 (COVID-19), which is caused by severe acute respiratory syndrome coronavirus 2 (SARS-CoV-2), the disease continues to spread worldwide. According to the COVID-19 weekly epidemiological update by the World Health Organization (WHO), almost 200 million confirmed cases and over 4.2 million documented deaths have been reported globally. Patients infected with SARS-CoV-2 show a wide range of symptoms and can be asymptomatic or have mild to severe disease symptoms requiring hospitalization. The severity of the symptoms results in both direct viral damage as well as unregulated inflammation called a cytokine storm^1^. Extensive global efforts have been made to combat COVID-19, which has led the approval of at least six prophylactic vaccines, including mRNA, viral-vector, and inactivated vaccines^2,3^. In addition, therapeutic agents such as antivirals, immunomodulators, and neutralizing agents are being developed.

Although considerable efforts are being made to develop prophylactic and therapeutic agents to combat COVID-19, genetic variants of SARS-CoV-2 have emerged and circulated worldwide. Since late 2020, several variants have emerged that pose an increased risk to global public health. Therefore, the WHO has defined specific variants as variants of interest (VOI) or variants of concern (VOC). Global monitoring, characterization, and research of VOCs have been prioritized for several reasons. For example, VOCs have shown evidence of an increase in transmissibility. In addition, there are possibilities of increasingly severe disease symptoms leading to an increase in hospitalizations or deaths, a substantial reduction in neutralization by antibodies generated during previous infections or vaccinations, reduced effectiveness of treatments or vaccines, or failures in diagnostic detection. As of the end of July 2021, four variants, B.1.1.7, B.1.351, P.1, and B.1.1617.2, were registered as VOCs and named by the WHO as the alpha, beta, gamma, and delta variants, respectively.

Animal models are crucial in the development and validation of prophylactics and therapeutics, as well as in clarifying the mechanisms underlying COVID-19 pathogenesis. SARS-CoV-2 leads to morbidity in non-human primates, Syrian hamsters, and ferrets^4–10^. In contrast, commercially available laboratory strains of mice do not exhibit infection by early SARS-CoV-2 isolates. To overcome this limitation, heterologous expression of human angiotensin-converting enzyme 2 (ACE2) in mice or mouse adaptation of the virus have been addressed^11–18^. Studies of mouse-adapted mutants have shown that a single amino acid change within the receptor binding domain (RBD) of the viral spike protein may cause infectivity in mice^15–18^. One of these mutations, the N501Y mutation, was also found in three of the four VOCs, specifically the B.1.1.7, B.1.351, and P.1 variants. The B.1.351 variant must especially be monitored carefully because it is spreading in South Africa and has been reported to be partly resistant to neutralization by currently available vaccines^19^. These findings motivated us to infect commercially available wild-type mice with these N501Y mutation-carrying VOCs, including the B.1.351 variant.

In this study, we investigated whether the SARS-CoV-2 B.1.351 variant can infect wild-type laboratory mice (BALB/c and C57BL/6 J mice). We found that BALB/c mice were more susceptible to infection by the B.1.351 variant than C57BL/6 J mice; compared to their counterparts, BALB/c mice showed a higher viral load and more vigorous inflammatory responses. Importantly, aged BALB/c mice (aged > 6 months) died approximately 1 week following SARS-CoV-2 infection. Furthermore, in these critical cases, several pro-inflammatory cytokines and chemokines were produced in a massive and prolonged manner, and their production profiles were similar to those in patients with severe COVID-19. These results demonstrate that young and aged wild-type laboratory mice infected with the N501Y-carrying mutants, such as the SARS-CoV-2 B.1.351 variant, are useful in analyzing a wide range of mild to critical COVID-19 symptoms and provide insights into the pathogenesis of COVID-19 that may be used to develop prophylactic and therapeutic strategies.

## Results

### Wild-type laboratory mice are susceptible to infection with the SARS-CoV-2 B.1.351 variant

**Figure 1:**
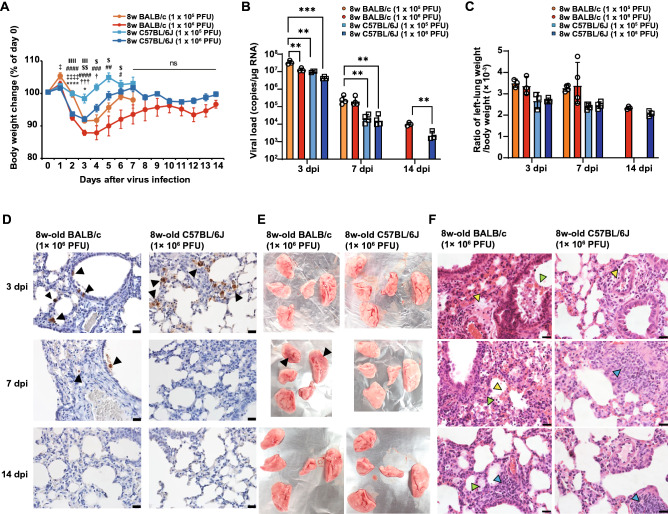
BALB/c mice showing severer symptoms than C57BL/6 J mice after infection by the SARS-CoV-2 B.1.351 variant (TY8-612 strain). (**A**) Mean body weight changes over 14 days after exposure to the TY8-612 strain. Each value represents a mean ± SEM of n = 3–10. *P* values were calculated via one-way-ANOVA followed by Tukey’s test, with *p* < 0.05 considered significant. Symbols indicate significant differences: *, between 1 × 10^5^ PFU of TY8-612-infected BALB/c and 1 × 10^6^ PFU of TY8-612-infected BALB/c. †, between 1 × 10^5^ PFU of TY8-612-infected BALB/c and 1 × 10^5^ PFU of TY8-612-infected C57BL/6 J. ‡, between 1 × 10^5^ PFU of TY8-612-infected BALB/c and 1 × 10^6^ PFU of TY8-612-infected C57BL/6 J. #, between 1 × 10^6^ PFU of TY8-612-infected BALB/c and 1 × 10^5^ PFU of TY8-612-infected C57BL/6 J. $, between 1 × 10^6^ PFU of TY8-612-infected BALB/c and 1 × 10^6^ PFU of TY8-612-infected C57BL/6 J. ‖, between 1 × 10^5^ PFU of TY8-612-infected C57BL/6 J and 1 × 10^6^ PFU of TY8-612-infected C57BL/6 J. (**B**) Viral loads of lung homogenates determined via RT-qPCR for the SARS-CoV-2 N protein gene in 3–4 mice at each time point following intranasal infection with the TY8-612 strain (1 × 10^5^ or 1 × 10^6^ PFU/head). *P* values were calculated via two-tailed unpaired one-way-ANOVA followed by Tukey’s test. (**C**) Ratio of wet left-lung weight to the initial bodyweight (day 0) compared among groups. (**D**–**E**) Data of 8-week-old BALB/c and C57BL/6 J mice infected with 1 × 10^6^ PFU of the T Y8-612 strain. (**D**) Detection of virus-infected cells in the lungs at 3, 7, or 14 dpi (SARS-CoV-2 N protein [brown staining, original magnification 400 × , scale bar = 20 μm]). Black arrowheads indicate infected cells. Nuclei were stained with hematoxylin. (E) Lung macroscopic appearance at indicated days. Black arrowheads indicate lesions in dark red. (**F**) Representative lung sections (hematoxylin and eosin staining, section thickness of 4 μm) from 8-week-old BALB/c and C57BL/6 J mice at 3, 7 or 14 dpi. The original magnification is 400 × for all micrographs (scale bar = 20 μm). Yellow arrowheads indicate perivascular edema (upper panels), hemorrhage, and congestion (left middle panel). Light green arrowhead indicates activated macrophage infiltration (left panels). Light blue arrowheads indicate lymphoid infiltration (left lower, right middle, and right lower panels).

Three of the four VOCs had the N501Y mutation in the RBD of the S protein. This mutation may confer the ability to bind to mouse ACE2, as one of the recently reported mouse-adapted mutants has an identical mutation, being the only mutation on its spike protein^17^. Thus, we investigated whether a circulating SARS-CoV-2 variant carrying the N501Y mutation on the S protein can infect wild-type laboratory mice, similar to a mouse-adapted mutant carrying the same mutation. Eight-week-old BALB/c and C57BL/6 J mice were infected intranasally with either 1 × 10^5^ or 1 × 10^6^ PFU of the TY8-612 strain of the B.1.351 variant of SARS-CoV-2. Body weight changes were monitored for seven days post infection (dpi) for animals infected with 1 × 10^5^ PFU of the TY-612 strain and for 14 dpi for animals infected with 1 × 10^6^ PFU of the TY-612 strain. As shown in Fig. 1A, both BALB/c and C57BL/6 J mice showed transient body weight loss in an infectious dose-dependent manner. For both infectious doses of the SARS-CoV-2 TY8-612 strain, body weight loss was greater in BALB/c mice than in C57BL/6 J mice. BALB/c mice infected with 1 × 10^6^ PFU of TY8-612 strain exhibited obviously ruffled fur at around 4 dpi but recovered this feature with their body weight gain. We measured viral loads in the left lung lobe of the infected mice at 3, 7, and 14 dpi via reverse transcription-quantitative PCR (RT-qPCR) to detect the nucleocapsid gene of SARS-CoV-2 (Fig. 1B). Interestingly, at 3 dpi, the viral loads in BALB/c mice infected with 1 × 10^5^ PFU were the highest among all the infected groups. At 7 dpi, the viral loads in BALB/c mice were substantially higher than those in C57BL/6 J mice. The higher viral loads in BALB/c mice were maintained up to 14 dpi. Similar to the viral RNA copy number results, the infectious viral loads at 3 dpi were mostly higher in both mouse strains infected with 1 × 10^5^ PFU of the TY-612 strain than in those infected with 1 × 10^6^ PFU of the TY-612 strain. Infectious viruses were undetectable 7 dpi or later, except for one BALB/c mouse and one C57BL/6 J mouse infected with 1 × 10^5^ PFU at 7 dpi (Supplementary Fig. 1A). Consistent with the viral load data, in BALB/c mice, N antigens were detected in bronchial epithelial cells, macrophages, and stromal cells in the sections of the right upper lung lobe via immunohistochemical staining at 3 and 7 dpi (Fig. 1D, left upper and left middle panels). In contrast, in C57BL/6 J mice, N antigens were detected in bronchial epithelial cells, macrophages, and alveolar epithelial cells at 3 dpi (Fig. 1D, right upper panel) but not at 7 dpi (Fig. 1D, right middle panel). The left lung lobes of the infected mice were weighed, and the ratio of the left lung lobe weight to the body weight prior to infection (day 0) was calculated (Fig. 1C). Although there was no significant difference, the ratio was higher in BALB/c mice than in C57BL/6 J mice at 3 and 7 dpi. Dark red and brown lesions were observed on the surfaces of the right upper and left lung lobes in BALB/c mice infected with 1 × 10^6^ PFU of the TY8-612 strain at 7 dpi (Fig. 1E, left middle panel, black arrowhead). In hematoxylin and eosin (H&E) staining analyses (Fig. 1F), BALB/c mice showed more severe pneumonia than C57BL/6 J mice. At 3 dpi, larger perivascular edema in the area was observed in BALB/c mice compared to C57BL/6 J mice (Fig. 1F: left upper and right upper panels, yellow arrowheads). In addition, a massive infiltration of macrophages was observed in the bronchi of BALB/c mice (Fig. 1F, left upper panel, light green arrowhead). At 7 dpi, thickened alveolar walls with macrophage infiltration and hemorrhage were observed in the lungs of BALB/c mice. Conversely, an accumulation of lymphocytes around blood vessels was observed in the lungs of C57BL/6 J mice, but minimal macrophage infiltration and hemorrhages were observed (Fig. 1F, right middle panel). Pneumonia in both BALB/c and C57BL/6 J mice recovered considerably at 14 dpi, although an accumulation of lymphocytes, which may have represented possibly inducible bronchus-associated lymphoid tissue, was observed (Fig. 1F, bottom panels). These results demonstrated that, compared to C57BL/6 J mice, BALB/c mice were more susceptible to infection with the SARS-CoV-2 B.1.351 variant.

### Infection with the SARS-CoV-2 B.1.351 variant lethally affects aged BALB/c mice

**Figure 2:**
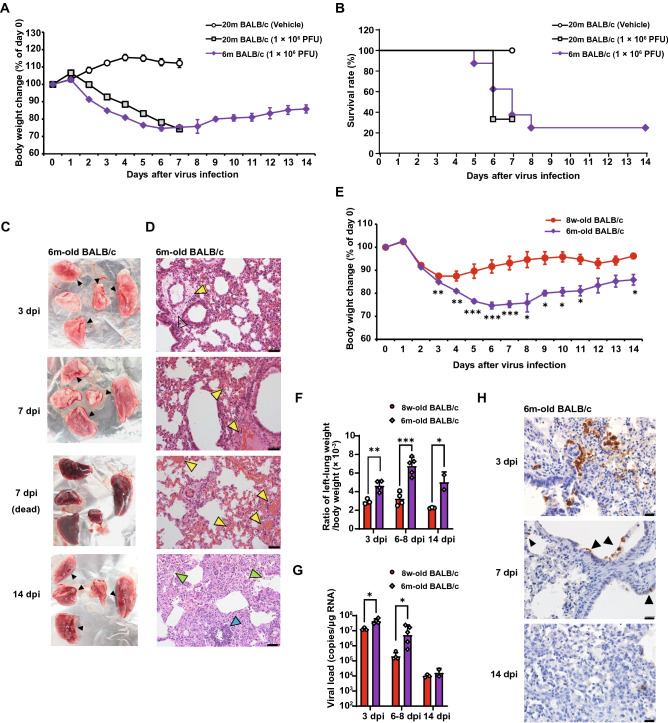
Aged BALB/c mice that succumbed to infection with the SARS-CoV-2 B.1.351 variant, with drastic weight loss, high viral replication, and severe pneumonia. Six-month-old (n = 12) and 20-month-old BALB/c mice (n = 3) were infected intranasally with 1 × 10^6^ PFU of the TY8-612 strain. Body weight was monitored daily (**A**). The survival rate was recorded until 7 dpi in 20-month-old BALB/c mice and 14 dpi in 6-month-old BALB/c mice (**B**). (**A**) Data are presented as mean ± SEM. (**C**) Lung macroscopic appearance on the indicated days. Black arrowheads indicate dark red lesions. (**D**) Representative sections (hematoxylin and eosin staining. section thickness: 4 μm) from 6-month-old BALB/c mice at 3, 7, or 14 dpi. For all micrographs, the original magnification was 200 × (scale bar = 50 μm). Yellow arrowheads indicate perivascular edema (top panel), hemorrhages and congestion (2nd and 3rd panels). Light green arrowheads indicate an activated macrophage accumulation (bottom panel). Light blue arrowhead indicates lymphoid infiltration (bottom panel). (**E**) Comparison of temporal changes in body weight between 8-week-old (young) and 6-month-old (aged) BALB/c mice. The body weight data of young and aged BALB/c mice were the same as those in Figs. 1B and 2A, respectively. Data are presented as mean ± SEM. P-values were calculated using a two-tailed unpaired Student’s *t*-test. (**F**) Ratio of wet left-lung weight per initial body weight (day 0) compared between young (n = 3–4 per time point) and aged (n = 2–5 per time point) groups. P-values were calculated using a two-tailed, non-paired Student’s *t*-test. (**G**) Viral loads of lung homogenates determined via RT-qPCR for the SARS-CoV-2 N protein gene and compared between young (n = 3–4 per time point) and aged (n = 2–5 per time point) groups. P-values were calculated using a two-tailed unpaired Student’s *t*-test or the Mann–Whitney U test. (**H**) Detection of virus-infected cells in the lungs at 3, 7, or 14 dpi (SARS-CoV-2 nucleocapsid protein [brown staining, original magnification 400 × , scale bar = 20 μm]). Black arrowheads indicate the infected cells. Nuclei were stained with hematoxylin.

Because aged BALB/c mice have showed an increased reaction severity to SARS^20,21^, we infected 20-month-old and 6-month-old BALB/c mice intranasally with 1 × 10^6^ PFU of the TY8-612 strain. Mock-infected 20-month-old BALB/c mice were inoculated intranasally with the same volume of vehicle (50 μL) as a negative control. Body weight changes and survival rates were monitored at 7 dpi in 20-month-old mice and 14 dpi in 6-month-old mice (Fig. 2A and B, respectively). The mock-infected 20-month-old BALB/c mice did not show any body weight loss and maintained a 100% survival rate during the experimental period (Fig. 2A and B). In contrast, following infection with the TY8-612 strain, both ages of BALB/c mice exhibited features such as ruffled fur, a hunched back, and reduced activity. Furthermore, more than 50% of the infected aged BALB/c mice exhibited diarrhea 5 dpi or later (data not shown), indicating that the TY8-612 strain caused multi-organ damage as well as respiratory symptoms. The 20-month-old BALB/c mice showed an irreversible and rapid decrease in body weight. Two-thirds of the infected 20-month-old mice died by the sixth day post-infection, and the remaining one mouse appeared emaciated at 7 dpi. The infected 6-month-old BALB/c mice showed a same or greater weight loss than the infected 20-month-old mice (Fig. 2A). Of the twelve 6-month-old mice, four were euthanized to collect organ samples and sera at 3 dpi; only two of the remaining eight mice survived to 14 dpi, except for one that was necropsied at 7 dpi. Hence, the survival rate was 25% (Fig. 2B). The two surviving mice did not regain weight during the experimental period. The macroscopic appearance of the lungs of the infected 6-month-old BALB/c mice at the indicated time points is shown in Fig. 2C. At 3 dpi, most of the lung lobes were already dark red in color (top panel in Fig. 2C, black arrowheads), suggesting either congestion or hemorrhages. The macroscopic discoloration of the lung lobes did not reverse, even at 14 dpi (the second and fourth panels from the top in Fig. 2C). In the dead mice, all lung lobes were reddish black in color (third panel from the top in Fig. 2C). Histopathological analyses demonstrated that massive perivascular edema occurred at 3 dpi, and congestion and hemorrhages occurred at 7 dpi (upper two panels in Fig. 2D). Congestion and hemorrhages were observed throughout all lung lobes in the animals that died at 7 dpi (third panel from the top in Fig. 2D, yellow arrowheads). At 14 dpi, accumulations of lymphocytes, granulation, and thickening of the alveolar wall caused by massive infiltration of macrophages were evident (bottom panel in Fig. 2D; light green arrowheads indicate macrophages, and the light blue arrowhead indicates the accumulation of lymphocytes).

Next, we compared body weight changes, the ratio of lung weight to body weight, and pulmonary viral loads of aged BALB/c mice (6 months) with those of young BALB/c mice (8 weeks) (Fig. 2E–G). Aged BALB/c mice showed significant body weight loss and increased lung weight compared to the young BALB/c mice. The pulmonary viral loads in aged BALB/c mice were significantly higher than those in young BALB/c mice at 3 and 7 dpi, whereas the viral loads were comparable between aged and young BALB/c mice at 14 dpi. Similarly, at 3 dpi, the infectious viral loads in the lungs of aged BALB/c mice were higher than those in the lungs of young BALB/c mice. Importantly, infectious viruses were detected in the lungs of all aged BALB/c mice at 6–8 dpi, thereby indicating that the virus can replicate in the lungs of aged animals longer than in younger specimens (Supplementary Fig. 1B). Consistent with the viral load data, N antigen-positive cells were observed at 3 and 7 dpi, but not at 14 dpi, even in lesions that accumulated inflammatory cells (Fig. 2H). These results demonstrated that 6-month-old BALB/c mice are highly vulnerable to infection with the TY8-612 strain and will exhibit lethal symptoms with high virus proliferation following infection.

### Expression levels of proinflammatory cytokines and chemokines following SARS-CoV-2 infection were massive and prolonged in critical cases in aged BALB/c mice

**Figure 3:**
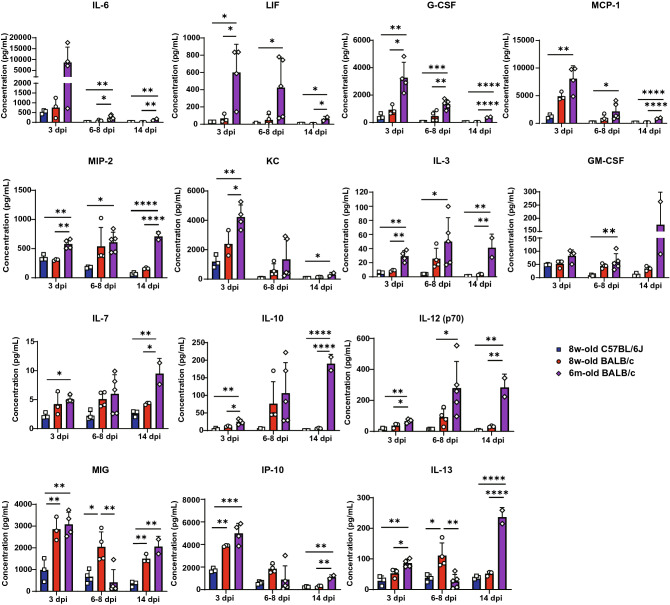
Cytokines and chemokines with the highest expression levels in aged BALB/c mice throughout the experimental period. Left-lung homogenates in HBSS were used to measure multiplex cytokines and chemokines using the Bio-plex suspension array system. Of the 32 cytokines and chemokines measured, 14 are shown in this figure. Data are presented as mean ± SD (young group, n = 3–4 per time point, aged group, n = 2–5 per time point). P-values were calculated using one-way ANOVA followed by Tukey’s test. Asterisks indicate significant differences among young C57BL/6 J, young BALB/c, and aged BALB/c mice.

**Figure 4:**
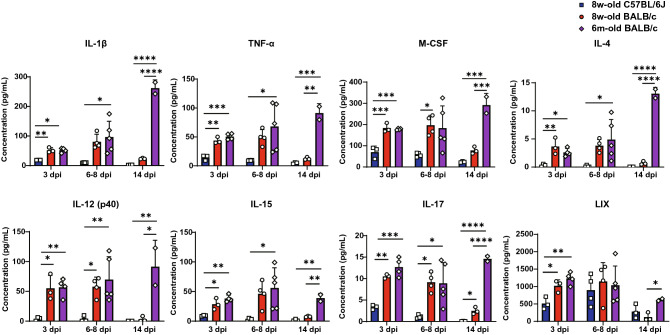
Cytokines and chemokines with higher expression levels in young and aged BALB/c mice compared to those in young C57BL/6 J mice from early to middle phase of SARS-CoV-2 infection. Left-lung homogenates in HBSS were used to measure multiplex cytokines and chemokines using the Bio-plex suspension array system. Of the 32 cytokines and chemokines measured, eight are shown in this figure. Data are presented as mean ± SD. (young group, n = 3–4 per time point; aged group, n = 2–5 per time point). P-values were calculated using one-way ANOVA followed by Tukey’s test. Asterisks indicate significant differences among young C57BL/6 J, young BALB/c, and aged BALB/c mice.

**Figure 5:**
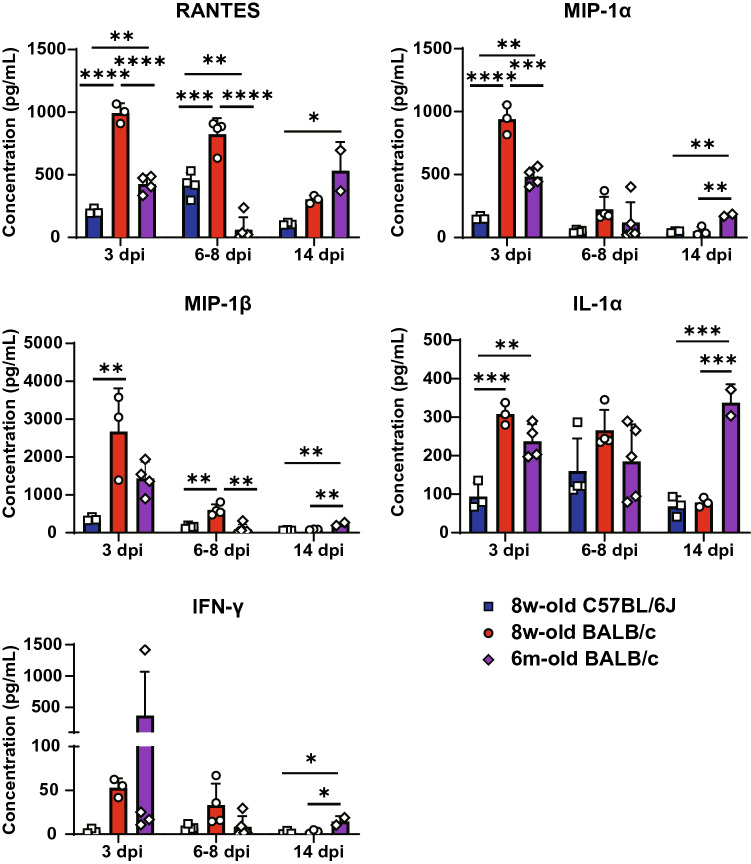
Cytokines and chemokines with the highest expression levels in young BALB/c mice from early to middle phase of SARS-CoV-2 infection. Left-lung homogenates in HBSS were used to measure multiplex cytokines and chemokines using the Bio-plex suspension array system. Of the 32 cytokines and chemokines measured, five are shown in this figure. Data are presented as mean ± S.D. (young group, n = 3–4 per time point, aged group, n = 2–5 per time point). P-values were calculated using one-way ANOVA followed by Tukey’s test. Asterisks indicate significant differences among young C57BL/6 J, young BALB/c, and aged BALB/c mice.

There was no significant difference in clinical illness between 6-month-old and 20-month-old BALB/c mice, including weight loss and lethality (Fig. 2A and 2B). This may indicate that 6-month-old BALB/c mice already have impaired immunological responses, which would thus increase COVID-19 reaction severity. We measured the levels of cytokines and chemokines in the lungs of young C57BL/6 J mice and young and aged BALB/c mice to identify the mediators involved in the differences in clinical illness observed between the groups. This was performed using a multiplex bead array that simultaneously detected 32 cytokines and chemokines. As shown in Fig. 3, the cytokines and chemokines with the highest expression levels in aged BALB/c mice during the infection interval were interleukin 6 (IL-6), leukemia inhibitory factor (LIF), granulocyte-colony stimulating factor (G-CSF), monocyte chemotactic protein 1 (MCP-1)/CCL2, macrophage inflammatory protein-2 (MIP-2)/CXCL2, keratinocyte-derived chemokine (KC)/CXCL1, IL-3, granulocyte macrophage colony-stimulating factor (GM-CSF), IL-7, IL-10, and IL-12 (p70 and mature dimers consisting of p35 and p40). In contrast, the expression levels of monokines induced by interferon gamma (MIG)/CXCL9, interferon gamma-induced protein 10 (IP10)/CXCL10, and IL-13 were highest in aged BALB/c mice at 3 dpi, but these values were highest in young BALB/c mice and low in weakened aged BALB/c mice at around 7 dpi. The lower expression levels of these IFN-γ-associated chemoattractants in aged BALB/c mice compared to those in young BALB/c mice can be attributed to the inefficient recruitment of antigen-specific CD8^+^ T cells to the lungs^22,23^. At 14 dpi, when the mice were recovering but still had severe pneumonia, the expression levels of these IFN-γ-associated chemoattractants increased again in aged BALB/c mice, thus suggesting that the migration and activation of macrophages and neutrophils may contribute to the critical illness in this model. Furthermore, the expression levels of IL-1β, tumor necrosis factor-alpha (TNF-α), macrophage colony-stimulating factor (M-CSF), IL-4, and IL-12 (p40), which are inducible subunits of IL-12, IL-15, IL-17, and liposaccharide-induced CXC chemokine (LIX)/CXCL5, were higher in both young and aged BALB/c mice than in C57BL/6 J mice from the early to middle phases of infection (Fig. 4). In addition, the expression levels of all cytokines and chemokines (shown in Fig. 4) were the highest in aged BALB/c mice at 14 dpi. Thus, these cytokines and chemokines were involved in prolonged inflammation in the lungs of aged BALB/c mice. As shown in Fig. 5, the expression levels of regulated on activation, normal T cell expressed and secreted (RANTES)/CCL5, macrophage inflammatory protein-1 alpha (MIP-1α)/CCL3, MIP-1β/CCL4, IL-1α, and IFN-γ (except for one aged BALB/c mouse in the early phase of infection) were highest in young BALB/c mice from the early to middle phases of infection. Reportedly, the responses of specific effector T cell populations generated during an acute pathogen challenge are partly controlled by several chemokines involved in chemotaxis^24,25^. This raises the possibility that the convergence of viral elimination and the inflammatory response was delayed, as shown in Fig. 2. The expression levels of Eotaxin/CCL11, IL-2, IL-5, IL-9, and the vascular endothelial growth factor (VEGF) were not significantly different among the three mouse groups throughout the experimental period, except that the expression levels of VEGF in aged BALB/c mice in the middle phase of infection were lower than those in young C57BL/6 J mice (Supplementary Fig. 2).

**Figure 6:**
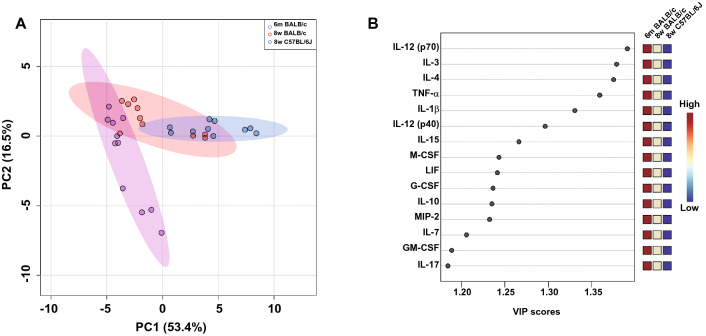
Principal component analysis (PCA) and the variable importance in projection (VIP) scores of lung-specific cytokines/chemokines induced in young C57BL/6 J mice as well as young and aged BALB/c mice following infection with the SARS-CoV-2 B.1.531 variant. Lung-specific cytokines and chemokines were subjected to PCA, and VIP scores were obtained from partial least squares-discriminant analysis (PLS-DA). (**A**) The two most significant principal components (PC1 and PC2) contained approximately 70% of the variance, which distinguished young C57BL/6 J mice and aged BALB/c mice based on the cytokine/chemokine profiles. Young BALB/c mice showed intermediate features compared to the other two groups. Each color ellipse represents a 95% confidence region. (**B**) VIP scores (top15) for cytokine/chemokine signatures facilitating the differences among young C57BL/6 J mice and young and aged BALB/c mice.

To extract features that would determine the symptom severity following SARS-CoV-2 infection, we performed principal analysis (PCA) for all 32 cytokines and chemokines in the lung homogenates measured in this study (Fig. 6A). The two most significant principal components (PC1 and PC2) explained approximately 70% of the total variation in the cytokine and chemokine data. This analysis showed that the cytokine and chemokine profiles of young C57BL/6 J mice were different from those of aged BALB/c mice, and that the profiles of young BALB/c mice were between those of the other two groups. We further conducted the variable importance project (VIP) to identify the cytokines and chemokines important in distinguishing the symptom severity among the three groups (Fig. 6B). The top 15 cytokines and chemokines were as follows: IL-12 (p70) (VIP score 1.39), IL-3 (VIP score 1.38), IL-4 (VIP score 1.37), TNF-α (VIP score 1.36), IL-1β (VIP score 1.33), IL-12 (p40) (VIP score 1.30), IL-15 (VIP score 1.27), M-CSF (VIP score 1.24), LIF (VIP score 1.24), G-CSF (VIP score 1.23), IL-10 (VIP score 1.23), MIP-2/CXCL2 (VIP score 1.23), IL-7 (VIP score 1.21), GM-CSF (VIP score 1.19), and IL-17 (VIP score 1.18). All the above cytokines and chemokines were expressed massively and persistently in aged BALB/c mice.

## Discussion

In this study, we obtained the following results: first, the SARS-CoV-2 B.1.351 variant infects wild-type laboratory mice, including BALB/c and C57BL/6 J mice. Second, BALB/c mice are more susceptible to infection with this variant than B6 mice, and exhibit worse symptoms, including greater body weight loss, higher viral proliferation, and higher cytokine/chemokine production (in terms of both type and quantity). Finally, and most importantly, infecting aged BALB/c mice with this variant leads to mortality.

Early SARS-CoV-2 isolates cannot easily infect wild-type laboratory mice because of the insufficient affinity of the virus for the mouse ortholog of the human receptor ACE2. Therefore, several reports have been published on the establishment of mouse-adapted mutants of SARS-CoV-2 with sufficient affinity for mouse ACE2 to infect wild-type mice^15–18^. Gu et al. reported that the resultant mouse adapted strain (called MASCp6) has the N501Y mutation at the RBD of the spike protein and shows increased infectivity in mouse lungs, leading to interstitial pneumonia and inflammatory responses in both young and aged mice^17^. More recently, the same group generated a lethal mouse-adapted strain of SARS-CoV-2 (MASCp36) in aged BALB/c mice with 30 passages of MASCp6 in murine lungs^26^. The MASCp36 strain has triple mutations at N501Y/Q493H/K417N in the spike protein. Of the triple mutations, N501Y and K417N are also included in the B.1.351 variant of SARS-CoV-2 and may support binding to murine ACE2. In addition to the B.1.351 variant, several SARS-CoV-2 variants carrying the N501Y mutation are currently prevalent worldwide, including the B.1.1.7 and P.1 variants. Because these other variants can also infect wild-type mice, further studies are necessary to analyze the infectivity and pathogenicity of other variants in wild-type mice. If these N501Y variants can be used to infect wild-type mice, in addition to improving the efficiency of animal experiments, they will also enable the use of a variety of genetically modified mice. Consequently, these improvements can be used to more rapidly detail the pathological mechanisms of COVID-19, the pathological differences of each variant, and the development of therapeutics and prophylactics.

In addition, the pathogenicity of VOCs in K18-hACE2 Tg mice has been reported, which is one of the most useful COVID-19 murine models. Bayarri-Olmos et al. showed that the alpha/B.1.1.7 variant of SARS-CoV-2 exhibits a significantly higher affinity for human ACE2 and causes faster disease progression and severity than an early 2020 SARS-CoV-2 isolate^27^. Radvak et al. demonstrated that SARS-CoV-2 alpha/B.1.1.7 and beta/B.1.351 variants are at least 100 times more lethal than the original SARS-CoV-2 strain. Young (8 to 10-week-old) K18-hACE2 mice infected with these two variants develop significant body weight loss, hypothermia, and severe internal organ damage ^28^. In contrast to K18-hACE2 mice, wild-type BALB/c mice were vulnerable to infection by the B.1.351 variant with aging. Namely, all 8-week-old BALB/c mice exhibited significant body weight loss, but recovered from challenge with the SARS-CoV-2 B.1.351 variant, whereas almost all aged BALB/c mice succumbed to the infection of B.1.351 at the same dose.

The genetic and phenotypic differences between BALB/c mice and C57BL/6 J mice may contribute to the observed differences in their symptoms. BALB/c mice preferentially develop a Th2 immune response, whereas C57BL/6 mice are Th1-dominant^29^. Macrophages from prototypical Th1 and Th2 strains of inbred mice are classified as M-1 and M-2 phenotypes and show different immunologic responses and phagocytic capacities^30,31^. However, M-1/M-2 phenotypes are independent of T or B lymphocytes, as these phenotypes are also observed in macrophages from C57BL/6 and BALB/c NUDE and SCID mice^30^. Alveolar macrophages (AMs) be integral in maintaining homeostasis and are considered the first line of host defense against respiratory microbes^32,33^. Su et al. demonstrated that the phagocytic capacity of M-1 AMs from C57BL/6 mice was greater than that of M-2 AMs from BALB/c mice^31^. C57BL/6 mice can likely clear viruses from the lungs more quickly and efficiently than their counterparts, leading to diminished inflammation. Consistent with this hypothesis, at 3 and 7 dpi, the viral loads in the lungs were lower in C57BL/6 J mice than in BALB/c mice (Fig. 1B). In contrast, BALB/c mice cleared the viruses slower, leading to a more intensive infiltration of monocytes and neutrophils and a greater inflammatory response.

Based on several reports that aged BALB/c mice have shown an increased reaction severity to SARS-CoV infection^20,21^, we demonstrated that BALB/c mice older than 6 months infected with SARS-CoV-2 TY8-612 strain showed rapid body weight loss and lethality of the infection. Regarding the susceptibility of aged mice to viral infections, Wong et al. found that the number of AMs essential for lung homeostasis decreases with aging, and that aging impairs the ability of AMs to limit lung damage, such as phagocytosis, during influenza virus infection^34^. Furthermore, in a study using MASCp36, a mouse-adapted SARS-CoV-2 strain described above, aged BALB/c mice succumbed to the virus infection through upregulating the transcription of a large number of cytokines and chemokines, extensive cell death, and alveolar type II epithelial (ATII) cell loss^26^. The authors also demonstrated that the ratio of ACE2^+^ ATII cells in uninfected aged mice was much higher than that in uninfected young mice, and that SARS-CoV-2-infected cells were more abundant in the lungs of aged mice than in young mice. Taken together, these results support the observed aged-skewed susceptibility to SARS-CoV-2.

Through analyzing cytokine and chemokine profiles in young C57BL/6 J mice and young and aged BALB/c mice following SARS-CoV-2 infection using PLS-DA and VIP scoring, we identified the cytokines and chemokines most implicated in symptom severity and show the top 15 most potent examples in Fig. 6B. The expression levels of these cytokines and chemokines were high and prolonged in aged BALB/c mice. The expression profiles of aged BALB/c mice are similar to those of patients with severe and critical COVID-19^35–38^. Among the proinflammatory mediators found elevated in infected aged BALB/c mice, many factors are associated with heart failure, resulting in congestion^39^. Accordingly, congestion was observed in all aged BALB/c mice after infection with the TY8-612 strain (Fig. 2C and D). There is a reportedly correlation between heart injury and COVID-19 severity^40^, thus suggesting that aged BALB/c mice are useful small animal models to investigate the mechanisms underlying heart injury in severe cases of COVID-19. Moreover, SARS-CoV-2 uses ACE2 as an entry receptor. ACE2 is expressed in the human vascular endothelium, respiratory epithelium, and other cell types^41^. Under physiological conditions, ACE2, via its carboxypeptidase activity, generates angiotensin fragments (Ang 1–9 and Ang 1–7) and is essential in the renin-angiotensin system (RAS), which is a critical regulator of cardiovascular homeostasis^42^. SARS-CoV-2 infection reduces ACE2 expression in lung cells^43^. Because the loss of the pulmonary ACE2 function is associated with acute lung injury, virus-induced ACE2 downregulation may be important in disease pathology. A reduction in ACE2 function following viral infection may result in RAS dysfunction, which influences blood pressure and fluid/electrolyte balance and enhances inflammation and vascular permeability in the airways. Using the SARS-CoV-2 N501Y-variant-infected aged BALB/c mouse model, the involvement of RAS in the severity of COVID-19 may be analyzed.

In contrast, the downregulation of RANTES/CCL5, MIP-1α/CCL3, and MIP-1β/CCL4 from the early to middle phase of the infection as well as the downregulation of MIG/CXCL9, IP-10/CXCL10, and IL-13 in the middle phase of the infection were observed in aged BALB/c mice. This raises the possibility that activating and recruiting antigen-specific CD8^+^ T cells is insufficient to control SARS-CoV-2 infection in the lungs because CD4^+^ T cells are inadequate^22–25^. This infection model will also allow us to analyze in detail the impact of antigen-specific T cell responses on the severity of COVID-19. In this study, the immune response analysis was limited to 32 cytokines and chemokines. It is possible that other mediators are involved in lethal phenotypes. Further studies using comprehensive analyses, such as proteomics and genomics, are required to identify other factors.

In conclusion, in this study, we established a COVID-19 murine model with various symptoms ranging from mild to moderate as well as critical conditions by infecting young B6 mice and young and aged BALB/c mice with the SARS-CoV-2 B.1.351 variant. The animals showed an increase in the magnitude and persistence of both viral replication and the production of cytokines/chemokines in correlation with the severity of their symptoms. These results demonstrate that wild-type mouse models infected with the SARS-CoV-2 B.1.351 variant are useful in preclinical testing of therapeutics and prophylactics. Furthermore, future studies using this model can also include detailed evaluations of immune responses and pathogenicity.

## Methods

### Ethics statement

All experiments using mice were approved by the Ethical Committee of Animal Experiments at the Tokyo Metropolitan Institute of Medical Science (Permission Number: 21–080). The experiments were performed in accordance with the Animal Experimentation Guidelines of the Tokyo Metropolitan Institute of Medical Science, the *Fundamental Guidelines for Proper Conduct of Animal Experiments and Related Activities in Academic Research Institutions* under the jurisdiction of the Ministry of Education, Culture, Sports, Science and Technology, Japan, and the ARRIVE guidelines.

### Viruses and cells

The SARS-CoV-2 TY8-612 strain (GISAID ID: EPI_ISL_1123289) of lineage B.1.351 was used in this study. This strain was provided by Dr. Masayuki Saijo, Mutsuyo Takayama-Ito, and Masaaki Satoh (Department of Virology 1, National Institute of Infectious Diseases), and was subcultured in Vero E6/TMPRSS2 cells (JCRB1819, Japanese Collection of Research Bioresources **(**JCRB) Cell Bank, National Institute of Biomedical Innovation, Health and Nutrition, Osaka, Japan) and grown in DMEM (Nissui Pharmaceutical Co. Ltd., Tokyo, Japan) supplemented with 10% inactivated fetal bovine serum, penicillin (100 units/mL), streptomycin (100 μg/mL), and G-418 (1 mg/mL)^44^. All procedures using SARS-CoV-2 were performed in biosafety level 3 facilities by personnel wearing powered air-purifying respirators (Shigematsu Co., Ltd., Tokyo, Japan).

### Plaque assay

The infectious SARS-CoV-2 titer was determined using a standard plaque assay. Briefly, the left lung lobe from each necropsied mouse was weighed and homogenized in nine volumes of Hanks-balanced saline solution or Leibovitz's L-15 medium (Thermo Fisher Scientific, Waltham, MA, USA) using the Multi-Beads Shocker (YASUI KIKAI, Osaka, Japan). Homogenates were centrifuged at 3000 × g for 10 min at 4ºC. The supernatant was collected and stored at − 80ºC until further use. Serially diluted (tenfold) supernatant or virus solutions (100 μL per well) were inoculated onto confluent monolayers of Vero E6/TMPRSS2 cells in 6-well plate and incubated at 37 °C for 1 h. Unbound viruses were removed by washing the cells with DMEM. The cells were then overlaid with DMEM containing 10% inactivated fetal bovine serum and 0.6% agarose (Sigma-Aldrich, St. Louis, MO, USA). After 48 h of incubation at 37 °C, the cells were fixed in 10% neutral buffered formalin and stained with 1% crystal violet. Data were acquired in two independent measurements, and their average is shown in the graph. The titer of SARS-CoV-2 was defined as plaque-forming units per gram of lung tissue (PFU/g lung) or PFU per milliliter (PFU/mL). The detection limit is 100 PFU/g lung for lung homogenates or 10 PFU/mL for virus stock solutions.

### Mouse study

BALB/c mice were purchased from SLC (Shizuoka, Japan), and C57BL/6 J mice were purchased from CLEA Japan, Inc. (Tokyo, Japan). The animals were given free access to food and water and were maintained on a 12 h light/12 h dark cycle. Prior to inoculation, the animals were anesthetized via intraperitoneal administration of a ketamine-xylazine mixture. The animals were inoculated intranasally with either 1 × 10^5^ PFU/50 µL or 1 × 10^6^ PFU/50 µL TY8-612 strain. Clinical signs were monitored for 14 dpi, and mice were euthanized at 3, 7, and 14 dpi to collect organ samples and sera. Body weight was monitored daily, and mice that lost 30% or more of their initial body weight were scored as dead and humanely euthanized. Changes in body weight were compared between young (3 dpi, n = 10, 4–7 dpi, n = 7, 8–14 dpi, n = 3) and aged (3 dpi, n = 12, 4–7 dpi, n = 4–8, 8–14 dpi, n = 2–3) BALB/c mice at their respective time points.

### Lung histopathology and immunohistochemistry

The upper lobe of the right lung from each necropsied mouse was fixed in 10% neutral buffered formalin, embedded in paraffin, sectioned at a 4 μm thickness, stained with H&E, and subjected to routine histological examination. Paraffine block sections were also used to stain the nucleocapsid (N) protein of SARS-CoV-2. Antigen retrieval was performed by autoclaving the sections in 10 mM citrate buffer (pH 6.0) for 10 min, and the sections were immersed in 0.3% hydrogen peroxide in methanol at room temperature (RT) for 30 min to inactivate endogenous peroxidase. The sections were blocked with BlockAce (DS Pharma Biomedical, Osaka, Japan) for 15 min and incubated overnight at 4 °C with 2 µg/mL of rabbit anti-N protein of the SARS-CoV-2 monoclonal antibody [HL344] (GTX635679, GeneTex, Inc., CA, USA). Secondary labeling was performed through incubation at RT for 30 min with EnVision + System-HRP labeled Polymer Anti-Rabbit (K4003; Dako Denmark A/S, Glostrup, Denmark), followed by color development with ImmPACT DAB Peroxidase Substrate (SK-4150; Vector Laboratories, Burlingame, CA, USA) at RT for 10 min. Nuclear staining was performed using a hematoxylin solution. Slides were imaged using an Axio Imager A2 microscope (Carl Zeiss Inc., Oberkochen, Germany).

### Viral RNA quantification

Total RNA samples were extracted from 70 μL of the supernatant of lung homogenates prepared for the plaque assay (refer subheading Plaque Assay) using RNeasy Mini kits (Qiagen, Hilden, Germany) according to the manufacturer’s instructions. Fifty nanograms of total RNA was used to quantify the gene of the SARS-CoV-2 N protein (http://www.cdc.gov/coronavirus/2019-ncov/lab/rt-pcr-panel-primer-probes.html): forward primer 5’-GACCCCAAAATCAGCGAAAT-3’ (2019-nCoV_N1-F), reverse primer 5’-TCTGGTTACTGCCAGTTGAATCTG-3’ (2019-nCoV_N1-R), and probe 5’-FAM-ACCCCGCATTACGTTTGGTGGACC-BHQ1-3’ (2019-nCoV_N1-P). Viral RNA was quantified using a 1-step reverse transcription-quantitative polymerase chain reaction (RT-qPCR) as previously described^6^. Viral loads were calculated as Log_10_ copies per microgram of total RNA.

### Multiple cytokine expression analyses

The supernatants of lung homogenates were assayed using the Bio-plex Suspension Array System, which utilizes luminex-based technology. For this study’s experiments, a mouse cytokine/chemokine magnetic bead panel (32-plex) was used according to the manufacturer’s instructions (Merck KGaA, Darmstadt, Germany).

### Statistical analyses

Data plotted on a linear scale are expressed as the mean ± standard deviation (SD), except for the mean ± standard error of mean (SEM) of body weight change. Data plotted on logarithmic scales are expressed as the geometric mean ± geometric SD. Inferential statistical analysis was performed using the two-tailed non-paired Student’s *t*-test, Mann–Whitney U test, or one-way ANOVA followed by Tukey’s test, as appropriate. Statistical significance was set at *p* < 0.05. The Prism software package (version 9.1; GraphPad Software) was used for all statistical analyses. Principal component analysis (PCA) and partial least squares-discriminant analysis (PLS-DA) were used to analyze cytokine/chemokine profiles among three different groups (young C57BL/6 J mice, young, and aged BALB/c mice). PCA plots were used to visualize if samples from different strains or aged mice following TY8-612 strain infection were separated into discrete groups within the two-dimensional principal component (PC) space. The variable importance in projection (VIP) scores indicate important features identified by PLS-DA. PCA, PLS-DA, and VIP were performed using MetaboAnalyst 5.0, which is a web-based platform for comprehensive data analysis and interpretation of metabolomics and other omics (https://www.metaboanalyst.ca/home.xhtml)^45^.

## Supplementary Information

Supplementary Information 1.

Supplementary Information 2.

## Data Availability

All other data are available from the corresponding author on reasonable request.
